# Combination of curcumin and piperine prevents formation of gallstones in C57BL6 mice fed on lithogenic diet: whether NPC1L1/SREBP2 participates in this process?

**DOI:** 10.1186/s12944-015-0106-2

**Published:** 2015-09-03

**Authors:** Yongnan Li, Min Li, Shuodong Wu, Yu Tian

**Affiliations:** Biliary & Vascular surgery, Shengjing Hospital of China Medical University, Shenyang City, 110004 PR China

**Keywords:** NPC1L1, SREBP2, Cholesterol absorption, Curcumin, Piperine, Gallbladder stone

## Abstract

**Background:**

A disruption of cholesterol homeostasis characterized by the physical-chemical imbalance of cholesterol solubility in bile often results in formation of cholesterol gallstones. Our earlier studies revealed that curcumin (1000 mg/kg) could prevent formation of gallstones. It has been proved that curcumin is poorly absorbed while piperine is a bioavailability-enhancer. Nevertheless, whether curcumin combined with piperine could enhance the effect of curcumin in preventing gallstones is still awaited.

**Method:**

C57BL6 mice were fed on a lithogenic diet concomitant with curcumin at 500 or 1000 mg/kg and/or piperine at 20 mg/kg for 4 weeks. The ratio of gallbladder stone formation was recorded and samples of blood, bile, gallbladder, liver and small intestine were also collected. The volume of gallbladder and weight of liver were calculated, and blood and bile samples were analyzed through biochemical methods. Intestinal NPC1L1 and SREBP2 mRNA and protein expression were detected by real-time PCR and Western blot.

**Result:**

Combining with piperine can significantly enhance the effect of curcumin, thus preventing the development of gallbladder stones, lowering the saturation of blood lipids and cholesterol in bile, as well as decreasing the expression of NPC1L1 and SREBP2 in both mRNA and protein levels.

**Conclusion:**

Curcumin can prevent the formation of cholesterol gallstones induced by high fat diet in mice and SREBP2 and NPC1L1 may participate in this process. Piperine can increase curcumin’s bioavailability, thereby enhancing the effect of curcumin.

## Background

A disruption of cholesterol homeostasis characterized by the physical-chemical imbalance of cholesterol solubility in bile often results in development of cholesterol gallstones. The homeostasis of cholesterol in human body mainly depends on its synthesis, absorption from intestine and secretion of the bile, of which the metabolic process is under precise regulation [1]. Previous studies have demonstrated that intestine is the unique organ providing dietary and reabsorbed cholesterol for the body, and the absorption of cholesterol often starts from the apical membrane of its epithelial cells. In light of the fact that there is a close relationship between cholesterol absorption and gallstone formation [2, 3], and clinical research has found that intracellular cholesterol transport was enhanced in patients with cholesterol gallstones [4]. So it seems that we can benefit a great deal by reducing the absorption of cholesterol from intestine to prevent gallbladder stones. Curcumin is the active ingredient in the traditional herbal remedy and dietary spice turmeric (*Curcuma longa*) [5]. And it has been researched in many directions, such as anti-carcinoma, anti-inflammatory, anti-oxidative as well as cardiovascular fields [6–10]. It has been reported that feeding lithogenic diet supplemented with 0.5 % curcumin for 10 weeks could reduce the incidence of gallstone formation to 26 % as compared to 100 % incidence in group fed with lithogenic diet alone in young male mice [11]. However, its low bioavailability, which due to poor absorption and faster metabolic alterations, presents a great challenge for its extensive applications [12–14]. Piperine, an alkaloid amide, is one of the major active components in black pepper. Suresh et al. [15] found that curcumin concomitant with piperine (20 mg/kg) not only reduced curcumin’s metabolic breakdown rate, which lead to prolonged retention of curcumin in the body, but also enhanced the intestinal absorption of curcumin. Kumar et al. [16] found that in Caco-2cells, curcumin could modulate Niemann-Pick C1-like 1 (NPC1L1) expression at transcriptional level with sterol response element-binding protein 2 (SREBP2) involved in the process. Moreover, our previous studies have indicated that 1000 mg/kg curcumin could prevent formation of cholesterol gallbladder stones. In this study, we attempted to find out whether lower dose of curcumin combined with piperine could have the same effect on gallstone-susceptible C57BL6 mice. Also, we explored if NPC1L1 and SREBP2 had participated in this process.

## Results

### Lipid levels of bile and blood

The serum concentration of cholesterol and triglyceride was significantly decreased in both LC500P20 and LC1000 compared with those in LD (*P* < 0.001). But no significant difference was found when mice were fed only with curcumin (500 mg/kg) or piperine (20 mg/kg) (Table 1)

**Table 1:** Lipid levels of bile and serum

	Bile(mmol/L)*			Serum(mmol/L) n = 10	
	Cholesterol	Phospholipid	Bile acid	Cholesterol	Triglyceride
CD	5.73	29.64	154.47	2.45 ± 0.31	0.27 ± 0.03
LD	18.64	28.93	138.64	6.86 ± 0.16**	1.59 ± 0.10**
LP20	15.92	29.79	143.24	5.80 ± 0.79***	1.56 ± 0.12
LC500	16.29	30.13	141.59	6.56 ± 0.42	1.58 ± 0.09
LC500P20	6.39	31.95	163.29	2.89 ± 0.14***	0.84 ± 0.10***
LC1000	6.59	32.24	161.35	2.96 ± 0.08***	0.84 ± 0.10***

*It was calculated by molarity, and the bile was collected for each experimental group

**P < 0.001 compared with CD

***P < 0.001 compared with LD

.

**Fig. 1:**
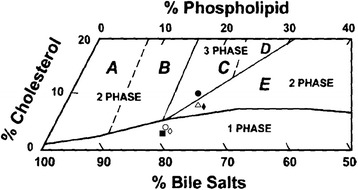
The relative lipid compositions of pooled gallbladder bile. ● represents relative lipid compositions of pooled gallbladder bile at 4 weeks in LD, Δ for LP20; ◆ for LC500; ◇ for LC500P20; ○ for LC1000; ▇ for CD

Bile cholesterol level (calculated by molarity) in LC500P20 and LC1000 decreased by over 60 % compared with those in LD, while phospholipid and bile acid varied slightly between each group. Moreover, the mole fraction of cholesterol, cholesterol/phospholipid ratio, as well as CSI values all decreased due to 1000 mg/kg curcumin or curcumin (500 mg/kg) administration combined with piperine (20 mg/kg) intervention (Table 2). The relative lipid composition of pooled gallbladder bile from mice in LD is located in the central three-phase zone, in which the bile is composed of mixed cholesterol monohydrate and lamellar liquid-crystalline pattern [17]. By contrast, the relative lipid compositions in LC500P20 and LC1000 are plotted in the one-phase micellar zone in which the bile is composed of unsaturated micelles at equilibrium (Fig. 1).

**Table 2:** Biliary lipid compositions of gallbladder bile

	Cholesterol	Phospholipid	Bile salt	Cholesterol		Total lipid concentration(g/dL)	CSI
	(Mole%^a^)	(Mole%^a^)	(Mole%^a^)	Phospholipid	Bile salt		
CD	3.02	15.61	81.37	0.20	0.04	10.1	0.546
LD	10.01	15.54	74.45	0.64	0.13	9.77	1.712
LP20	8.43	15.77	75.81	0.53	0.11	9.96	1.465
LC500	8.66	16.03	75.31	0.54	0.11	9.92	1.476
LC500P20	3.17	15.85	80.98	0.2	0.04	10.74	0.554
LC1000	3.29	16.11	80.6	0.20	0.04	10.68	0.575

^a^Mole fraction

### Prevention of cholesterol gallstones

Our previous research indicated that mice fed solely on lithogenic diet for 4 weeks showed 100 % formation of cholesterol gallstones. The reduced incidence of gallstones amounted to 30 %, 20 %, 70 % and 60 % respectively in corresponding groups compared with LD (Table 3). The volume of gallbladder increased when fed on lithogenic diet compared with CD, while showed slightly difference between each group with or without curcumin and/or piperine. In addition, the weight of liver decreased obviously when mice were fed on 1000 mg/kg curcumin or 20 mg/kg piperine or curcumin combined with piperine (Table 4).

**Table 3:** Prevention of cholesterol gallstones by curcumin and piperine

	Incidence of cholesterol gallstones(%)	Reduction in incidence of cholesterol gallstones(%)
CD	0	-
LD	100 %	-
LP20	70 %	30 %
LC500	80 %	20 %
LC500P20	30 %	70 %
LC1000	40 %	60 %

There are 10 mice per group

**Table 4:** Volume of gallbladder and weight of liver

	Gallbladder (*n* = 10)			Liver (*n* = 10)
	Length(mm)	Width(mm)	Volume(μL)	Weight(g)
CD	6.35 ± 0.91	2.75 ± 0.32	25.24 ± 5.97	0.90 ± 0.07
LD	8.19 ± 1.71	3.87 ± 0.48	65.54 ± 22.69*	1.93 ± 0.18*
LP20	7.36 ± 1.51	3.61 ± 0.70	53.90 ± 28.08	1.32 ± 0.18***
LC500	7.91 ± 0.87	3.66 ± 0.50	61.41 ± 18.44	1.90 ± 0.16
LC500P20	6.66 ± 1.36	3.09 ± 0.57	35.98 ± 15.27**	1.15 ± 0.19***
LC1000	7.18 ± 1.04	3.49 ± 0.72	49.05 ± 25.01	1.23 ± 0.13***

Values are means ± SD, with 10 samples each group

*P < 0.001 compared with CD, **P < 0.05 compared with LD, ***P < 0.001 compared with LD

### Histopathology of gallbladder and liver

**Fig. 2:**
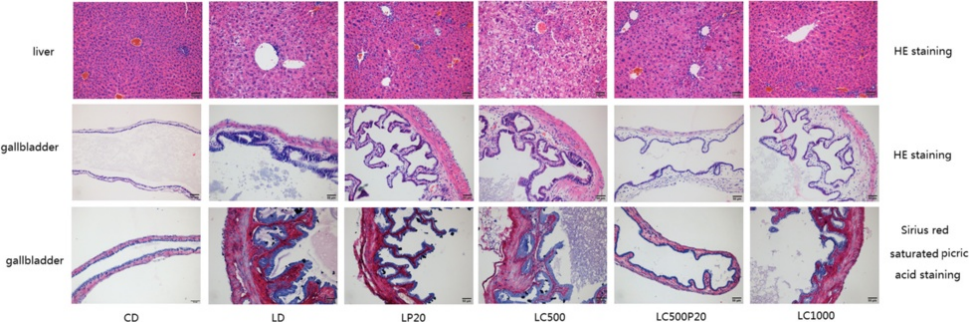
HE staining and sirius red saturated picric acid staining in liver and gallbladder

HE staining of liver revealed obvious vacuolar degeneration, coupled with neutrophils infiltrating into acini and portal areas in LD. By comparison, the changes mitigated with curcumin-piperine group. HE staining and sirius red saturated picric acid staining of gallbladder indicated mucosal hyperplasia and connective tissue expansion of lamina propria in LD, as well as a significant increase of small vessels and collagenous fibers. In contrast, these changes could be inhibited by curcumin combined with piperine (Fig. 2).

**Fig. 3:**
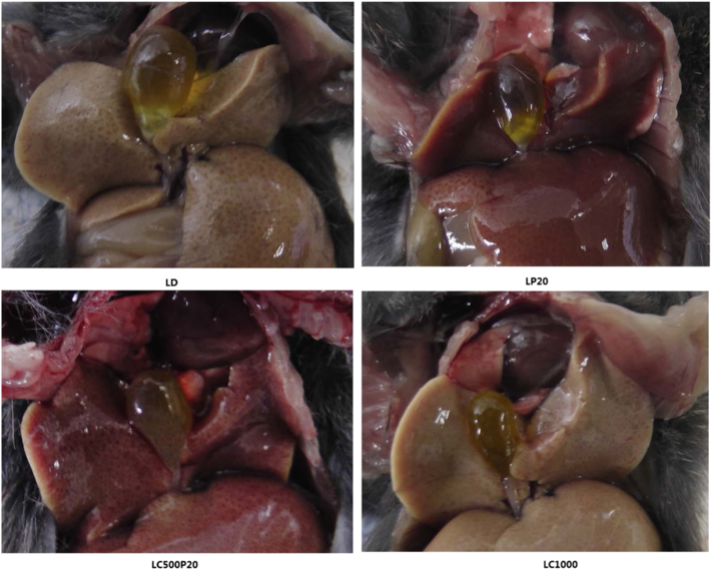
Changes of liver and gallbladder in different groups. LD: Liver underwent hepatic steatosis and gallbladder stones formed. LP20: Liver appeared normal, while gallbladder stones were observed. LC500P20: Liver appeared normal and no stones were seen in gallbladder. LC1000: Liver underwent fatty degeneration and no stones were observed

Interestingly, we found that livers of mice fed on piperine (concomitant with/without curcumin) showed a protective effect against lithogenic diet. Livers of mice in LD showed hepatic steatosis under light microscope, including hepatomegaly, yellowing of liver, edge blunting, greasy touch and vacuolar degeneration. While in piperine group, the color of liver turned red, its edge sharpened and cell arrangement became normal under light microscope (Fig. 3).

### Curcumin combined with piperine reduces NPC1L1 expression

**Fig. 4:**
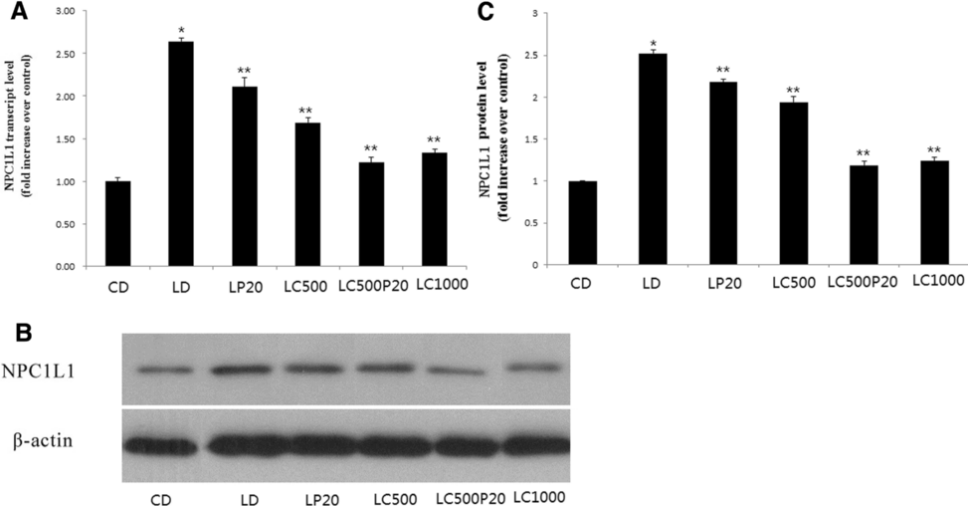
Curcumin combined with piperine reduces NPC1L1 expression. **a** NPC1L1 mRNA levels. **b** NPC1L1 protein expression. **c** Grey analysis for western blot

Both of the spice principles could inhibit NPC1L1 mRNA expression, especially when curcumin (1000 mg/kg) or curcumin combined with piperine (1.00 ± 0.04 vs 2.64 ± 0.04, 2.11 ± 0.11, 1.69 ± 0.06, 1.22 ± 0.06, 1.33 ± 0.05 respectively, *P < 0.001 compared with CD, **P < 0.001 compared with LD) were administered (Fig. 4a). Furthermore, results of Western blot revealed that 1000 mg/kg curcumin or curcumin combined with piperine could reduce the expression of NPC1L1 protein (Fig. 4b). Fig. 4c was grey analysis for western blot (1.00 vs 2.52 ± 0.038, 2.18 ± 0.036, 1.94 ± 0.072, 1.19 ± 0.05, 1.24 ± 0.049, *P < 0.001 compared with CD, **P < 0.001 compared with LD).

### Curcumin combined with piperine reduces SREBP2 expression

**Fig. 5:**
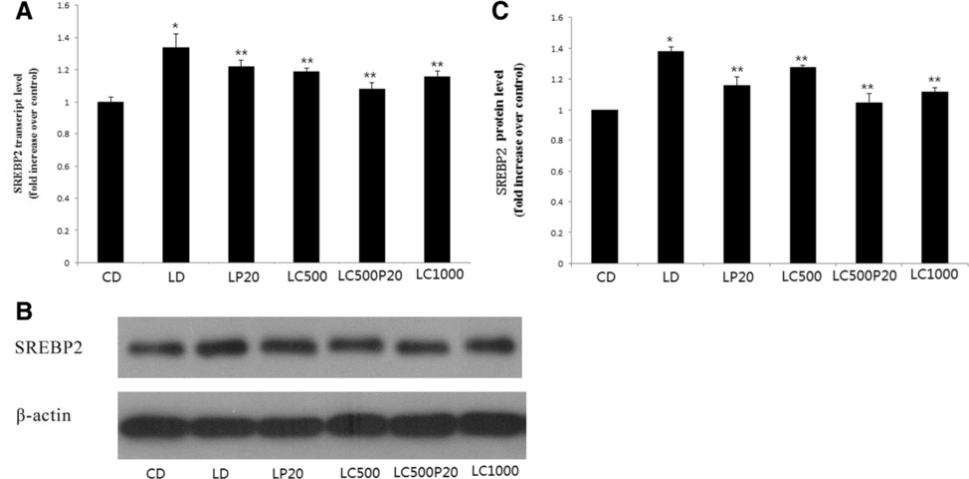
Curcumin combined with piperine reduces SREBP2 expression. **a** SREBP2 mRNA expression. **b** SREBP2 protein expression. **c** Grey analysis for western blot

Treatment with curcumin significantly inhibited SREBP-2 mRNA expression, especially when mice were fed on curcumin combined with piperine (1.0 ± 0.03 vs 1.34 ± 0.08, 1.22 ± 0.08, 1.19 ± 0.02, 1.08 ± 0.04, 1.16 ± 0.03 respectively, *P < 0.001 compared with CD, **P < 0.01 compared with LD) (Fig. 5a). We also found that curcumin combined with piperine could decrease SREBP2 protein expression as 1000 mg/kg curcumin did (Fig. 5b). Fig. 5c was grey analysis for western blot (1.00 vs 1.38 ± 0.03, 1.16 ± 0.05, 1.28 ± 0.01, 1.05 ± 0.05, 1.12 ± 0.03,*P < 0.001 compared with CD, **P < 0.01 compared with LD).

## Discussion

Curcumin is the active ingredient of traditional herbal remedy and dietary spice turmeric, and has a long history of use in traditional Chinese medicine [5]. Curcumin has been widely investigated of its numerous biological effects, including anti-carcinoma, anti-inflammatory and antioxidative effect as well as its efficacy in cardiovascular diseases [6–10]. Our previous study has evidenced that curcumin could prevent formation of gallstones in C57BL6 mice in a dose dependent manner, especially when taken at 1000 mg/kg. However, this curcumin intake is much more than our normal consumption in daily life. Besides, curcumin is poorly absorbed from intestine and can be rapidly metabolized in liver and intestinal wall [12–14]. So it has always been a challenge on how to enhance the oral bioavailability of curcumin. Then, the introduction of piperine cast a new light on this research.

Piperine is an alkaloid isolated from *Piper nigrum* fruits, and it is the first batch of purified natural molecules with bio-enhancer properties [18]. Piperine is pharmacologically safe and has been listed in compounds ‘generally regarded as safe’ according to the US Food and Drug Administration (FDA) [19]. It is also an inhibitor of hepatic and intestinal glucuronidation, and it has been reported that the ingestion of piperine contributed to increase the serum concentration of curcumin and thereby its bioavailability [20, 21]. Shoba et al. [22] have found that concomitant administration of piperine 20 mg/kg could increase curcumin’s (2 g/kg) bioavailability by 154 % in rats and 2000 % in human. Later, Suresh et al. [15] found 500 mg/kg curcumin combined with 20 mg/kg piperine could made an increase of curcumin absorption from 60 %–66 % to 78 % in experimental animals. Moreover, Singh et al. [23] found that piperine might enhance the bioabailability of curcumin through P-glycoprotein. P-glycoprotein is organized in two homologous halves, each half begins with a transmembrane domain that containing six transmembrane segments followed by a hydrophilic nucleotide-binding domain [24]. Therefore, we adopted curcumin (500 mg/kg) in combination with piperine (20 mg/kg) for our research, and found that curcumin combined with piperine could lower serum cholesterol and triglyceride, which was more effective than LC500, and similar with LC1000 (Table 1). It is similar with study of Tu et al. [25], who found that curcumin plus piperine could decrease levels of total cholesterol (TC), triglyceride (TG) and low-density lipoprotein in serum, as well as increase levels of fecal TC, TG and total bile acid compared with administration of curcumin alone. It was found that piperine alone also could decrease serum cholesterol, though not as obviously as curcumin combined piperine did (Table 1). Duanqjai et al. [26] found piperine could reduce cholesterol uptake by internalizing the cholesterol transporter protein, thus, reduced serum cholesterol. Curcumin combined with piperine also made cholesterol settled out (Table 2 and Fig. 1), and finally, inhibited formation of gallstones, while piperine taken alone did not have the effect of preventing gallstones (Table 3). It has been reported that curcumin has an effect of liver protection [6], and our previous studies indicated that the liver weight of mice fed on lithogenic diet and curcumin was significantly decreased in a dose dependent manner, compared with those from LD. However, even for mice fed on 1000 mg/kg curcumin, hepatic steatosis was still observed in progress. In this study, we were surprised to find that piperine had a significant effect on hepatoprotection. Liver of mice in LD showed a typical hepatic steatosis, including hepatomegaly, which include yellowing of liver, edge blunting, greasy touch as well as vacuolar degeneration under light microscope. When fed on piperine (with or without curcumin), the color of liver turned red, its edge sharpened and cell arrangement became normal (Fig. 3). It is consistent with the perspective of Hyejeong et al. [27] who thought piperine could alter liver X receptor α (LXRα)-mediated lipogenesis, including SREBP1c. Moreover, Seoyoon et al. [28] reported that the effect of piperine on hepatic steatosis was probably due to reduced expression of genes involved in lipogenesis, as well as enhanced expression levels of genes involved in fatty acid oxidation mediated by adiponectin-AMPK pathway. Therefore, the mechanism of piperine on liver-protection deserves more intensive study and may provide a new insight into prevention and treatment of fatty livers. Interesting, co-administration of curcumin with piperine seemes could not bring advantage to the curcumin effects on antidiabetic and antioxidant activities, which Carlos et al. [29] suspected might be related to changes on its biotransformation. Therefore, at least part of the curcumin actions may be related to metabolites.

Homeostasis of cholesterol in human body mainly depends on its synthesis, absorption from intestine and secretion of the bile, the metabolic process of which is under precise regulation [1]. Previous studies have revealed that intestine is the unique organ providing dietary and reabsorbed cholesterol for the body, and the absorption of cholesterol often starts from the apical membrane of epithelial cells. Besides, there is a close relationship between cholesterol absorption and gallstone formation [2, 3]. There is also evidence that deletion of Niemann-Pick C1-like 1(NPC1L1) gene or use of its specific inhibitor, ezetimibe, could decrease up to 70 % of cholesterol absorption in intestine [30–32]. Our earlier studies found that curcumin could decrease NPC1L1 mRNA and protein expression in a dose-dependent manner. While in this study, it was found that 20 mg/kg piperine or curcumin combined piperine could significantly decrease expression of NPC1L1 mRNA and protein expression, especially in LC500P20, which showed even lower expression than LC1000. The expression of NPC1L1 is regulated by SREBP2 [33] and it has been reported that the -291/+56 region of the NPC1L1 gene harbors a binding site for the SREBP2, which is essential for the basal activity of the NPC1L1 promoter as well as cholesterol modulation [34, 35]. Earlier studies from our laboratory demonstrated that curcumin could decrease SREBP2 mRNA and protein expression in a dose-dependent manner. In this research, it was found that both piperine and curcumin combined piperine could decrease SREBP2 mRNA and protein expression, especially when applied in combination, the inhibition rate was superior to that of 1000 mg/kg curcumin group.

## Conclusion

In summary, our present study demonstrated that curcumin can prevent formation of gallstones, and it is likely due to reduced expression of NPC1L1 that regulated by SREBP2. Moreover, piperine, as an enhancer of curcumin, could increase curcumin bioavailability and make curcumin more effective in preventing gallstones. Since curcumin is easy to access in daily life and piperine is ‘generally regarded as safe’ by FDA, more research on curcumin combined with piperine deserves to be carried out to bring new insight into maintaining cholesterol homeostasis in our daily diet.

## Materials and methods

### Animals and diets

Male C57BL6 mice, 6-8 weeks old, were purchased from Central Laboratory of Shengjing Hospital. Mice were fed on normal rodent feedstuff (cholesterol < 0.02 %) or a lithogenic diet [36] (2 % cholesterol plus 0.5 % cholic acid and 15 % buffer) for 4 weeks, and divided into six groups (10 mice/group): (1) Normal rodent feedstuff(Control diet, CD), (2) Lithogenic diet(LD), (3) Lithogenic diet + 20 mg/kg piperine(LP20), (4) Lithogenic diet + 500 mg/kg curcumin(LC500), (5) Lithogenic diet + 500 mg/kg curcumin + 20 mg/kg piperine(LC500P20), (6) Lithogenic diet +1000 mg/kg curcumin(LC1000). Mice of all groups (except CD and LD) were treated by gavage with curcumin/piperine for 4 weeks after weighed every day at 09:00. The curcumin, piperine and both were administered in 1 ml 0.5 % sodium carboxymethyl cellulose. Mice of CD and LD group received 1 ml 0.5 % sodium carboxymethyl cellulose every day. All mice in six groups got free access to water, and were kept under controlled condition at room temperature (22 ± 3 °C), with a relative humidity of 60 %–70 % and a 12-h exchange of light/dark cycle. The animal experiments were conducted according to the regulations of the Committee on Bioethics of China Medical University.

### Collection of gallbladder, liver and small intestine

After the last gavage, all mice were fasted for 12 h and then anesthetized using 10 % chloral hydrate. Blood was drawn immediately through cardiac puncture and serum was prepared by centrifugation. Each size of gallbladder was measured by vernier caliper. Cholecystectomy was performed and gallbladder was cautiously removed with its adhering tissues being cleared up. Bile was collected from each experimental group and stored at -80 °C for subsequent extraction and analysis. Livers were excised, washed with 0.9 % saline, blotted dry and weighted, then they were stored at -80 °C for extraction and analysis. Finally, a section of small intestine was resected (about 4–5 cm) from each mouse.

### Lipid analysis

Levels of bile cholesterol, phospholipid, bile salts as well as cholesterol and triglyceride in serum were determined respectively following manufacturer’s instructions. Cholesterol saturation index (CSI) of the bile was calculated according to Carey tables [37].

### Histopathology

Paraffin-embedded gallbladder and liver sections (5 μm in thickness) were used for hematoxylin and eosin staining (HE staining). They were observed at 200× magnification under light microscope.

1 % sirius red saturated picric acid staining was used for paraffin-embedded gallbladder sections (5 μm in thickness). The sections were observed at 400× magnification under light microscope.

### Real-time PCR analysis

Total RNA was isolated using Trizol reagent (Takara) following manufacturer’s instructions, the concentration of RNA was calculated by spectrophotometry. cDNA was prepared using the PrimeScript RT reagent kit with gDNA Eraser (Takara). Real-time PCR was performed using SYBR Green Premix Ex Taq (Takara). Sequences for the primers used were as follows:

- NPC1L1: (forward) 5’-GACATCACCTTCCACCTCTTG-3’,
- (reverse) 5’- CTGGCATTCGACCCATGTAG-3’
- SREBP2: (forward) 5’-TGGGGACAGATGCCAAGATG-3’,
- (reverse) 5’- CACCAGACTGCCCAAGTCGA-3’
- β-actin: (forward) 5’-CTGTGCCCATCTACGAGGGCTAT-3’,
- (reverse) 5’- TTTGATGTCACGCACGATTTCC-3’

For real-time PCR, the PCR mixture was denatured at 95 °C for 10s, annealed at 60 °C for 20s and then extended at 72 °C for 30s. This process was repeated for a total of 40 cycles. The relation of NPC1L1 and SREBP2 mRNA expression with β-actin was calculated basing on the threshold cycle (Ct) values.

### Western blot analysis

Protein concentration was measured using the bicinchoninic acid (BCA) protein assay. Protein samples were separated on 8 % SDS-PAGE gels and transferred to polyvinylidene difluoride membranes (Millipore, USA). Non-specific binding to the membrane was blocked for 1 h at room temperature with 5 % fat-free milk in TBST, and then the membranes were incubated with 1:2000 NPC1L1 primary antibody (Novus Biologicals, USA) and 1:2000 SREBP2 primary antibody (Abcam) respectively at 4 °C overnight. Then, the membrane was washed four times with TBST and incubated with a 1:5000 dilution of the appropriate secondary antibody at room temperature for 45 mins. After the membrane was washed twice with TBST, membrane-bound antibody was visualized using an enhanced chemiluminescent kit (Millipore) according to manufacturer instructions.

### Statistical analysis

Values are given as the mean ± SD. Differences between multiple groups were compared using one-way analysis of variance (ANVOA). When statistical significance was identified based on ANOVA, the Student-Newman-Keuls test was used for multiple comparisons. P-values < 0.05 were regarded as statistically significant.

## Abbreviations

NPC1L1: Niemann-Pick C1-like 1
SREBP2: Sterol response element-binding protein 2
TC: Total cholesterol
TG: Triglyceride
